# Alzheimer's Disease and Type 2 Diabetes: A Critical Assessment of the Shared Pathological Traits

**DOI:** 10.3389/fnins.2018.00383

**Published:** 2018-06-08

**Authors:** Shreyasi Chatterjee, Amritpal Mudher

**Affiliations:** Centre of Biological Sciences, University of Southampton, Southampton, United Kingdom

**Keywords:** insulin resistance, tau proteins, abeta oligomers, synaptic dysfunction, autophagy, inflammation

## Abstract

Alzheimer's disease (AD) and Type 2 Diabetes Mellitus (T2DM) are two of the most prevalent diseases in the elderly population worldwide. A growing body of epidemiological studies suggest that people with T2DM are at a higher risk of developing AD. Likewise, AD brains are less capable of glucose uptake from the surroundings resembling a condition of brain insulin resistance. Pathologically AD is characterized by extracellular plaques of Aβ and intracellular neurofibrillary tangles of hyperphosphorylated tau. T2DM, on the other hand is a metabolic disorder characterized by hyperglycemia and insulin resistance. In this review we have discussed how Insulin resistance in T2DM directly exacerbates Aβ and tau pathologies and elucidated the pathophysiological traits of synaptic dysfunction, inflammation, and autophagic impairments that are common to both diseases and indirectly impact Aβ and tau functions in the neurons. Elucidation of the underlying pathways that connect these two diseases will be immensely valuable for designing novel drug targets for Alzheimer's disease.

## Introduction

### Alzheimer's disease: neuropathological alterations and metabolic risk factors

Diagnosed by the German psychiatrist and neuropathologist, Prof. Alois Alzheimer in 1906, Alzheimer's disease is the most prevalent form of dementia in the aging population (van der Flier and Scheltens, 2005). Recently declared as the sixth major cause of death in the world, patients affected with AD suffer a gradual decline of cognitive abilities and memory functions till the disease renders them incapable of performing daily functions (James et al., 2014). Statistical data reveals that over 30 million people are suffering from AD worldwide and this number is estimated to double every 20 years to reach 66 million in 2030 and about 115 million by 2050^1^.

Clinically AD can be classified into two subtypes. About 95% 0f AD patients are aged 65 years or older and are diagnosed with “late-onset” or “sporadic AD” (sAD) while 5% of AD patients carry rare genetic mutations associated with “early-onset” or “familial AD” (fAD) that causes the onset of disease symptoms in a person's thirties, forties, or fifties (De Strooper, 2007). In early onset fAD, the disease pathology is caused by mutation in three known genes namely: amyloid precursor protein (APP), presenilin-1 (PS-1), and presenilin-2(PS-2). Although PS-1 mutations account for most of the fAD, there are mutations outside these three genes that are yet unknown. Unlike the fAD, the genetics of sAD is more complex (Dorszewska et al., 2016). Other than aging, which is the strongest risk factor for sAD, GWAS studies reveal that the epsilon four allele of the apolipoprotein E (ApoE4) gene is a significant risk factor for the development of this disease. Two copies of ApoE4 gene increases risk of AD by 12-fold, while one copy of this allele enhances the risk by 4-fold (Bertram and Tanzi, 2009). However, only 50–60% individuals are carriers of this gene suggesting that other factors also confer risk. Studies suggest that these include factors such as cerebrovascular infarction, family history of diabetes, hypertension, and obesity (Li et al., 2015a).

At a cellular level, AD is characterized by a progressive loss of pyramidal cells in the entorhinal cortex and CA1 region of the hippocampus that are responsible for maintenance of higher cognitive functions (Serrano-Pozo et al., 2011). Early symptoms of AD are also marked by synaptic dysfunction that disrupts connectivity between neural circuits, thereby initiating the gradual loss of memory.

Neuropathologically, AD is characterized by extracellular plaques of insoluble amyloid-β protein, and intracellular neurofibrillary tangles (NFTs) of hyperphosphorylated tau protein (Iqbal et al., 2010; Serrano-Pozo et al., 2011). In AD, abnormal cleavage of APP results in the formation of insoluble amyloid-β protein, densely packed with beta sheets, which form the core of the senile plaques (Serpell et al., 2000). Tau protein, in a physiological state serves as a microtubule binding protein and plays an important role in axonal and vesicular transport (Mandelkow and Mandelkow, 1995). Conversely, in the disease state tau protein is hyperphosphorylated and detached from the microtubules. In animal models this phospho-tau-mediated disruption of cytoskeletal integrity manifests in synaptic and behavioral impairments (Mudher et al., 2004; Quraishe et al., 2013; Gilley et al., 2016; Lathuilière et al., 2017). Although a large body of *in vitro* studies have investigated tau-microtubule binding interactions, most of these studies have been conducted *in silico* or in non-neural cellular models (Kadavath et al., 2015; Huda et al., 2017). In a series of elegant experiments involving single-molecule tracking of tau in axonal processes, Niewidok et al. and Janning et al. have shown that the interaction of tau with the microtubules follows a “kiss-and-hop” mechanism. Their studies show that a single tau molecule resides only 40 ms on a particular microtubule and then hops longitudinally and transversely on adjacent microtubules. This novel mechanism has been particularly effective in resolving the paradoxical observation that despite regulating microtubule dynamics, alterations in tau levels may not interfere with axonal transport (Janning et al., 2014; Niewidok et al., 2016). Using pseudo-hyperphosphorylated tau constructs they observed a considerable weakening of the tau-microtubule interactions that corroborated with previous *in vitro* studies.

A large body of evidence supports the idea that the formation of Aβ plaques occurs 15–20 years earlier before the cognitive functions decline, whereas the spatial and temporal spread of tau pathology correlates more strongly with the severity with disease progression (Serrano-Pozo et al., 2011; Vlassenko et al., 2012). Although Aβ and tau are the pathological hallmarks that characterize sAD, it is not yet clear whether these two factors trigger AD or if they are manifested as the effect of the disease.

The drug therapy of AD is still at a nascent stage providing symptomatic relief but not slowing down disease progression. Such treatments include FDA-approved choline esterase inhibitors and NMDA (glutamate) receptor agonists. Thus, from a public health perspective AD exerts a significant healthcare burden that is expected to escalate 5-fold in the coming decades. Hence, the need for early detection and effective treatment is an urgent priority (Yiannopoulou and Papageorgiou, 2013).

In recent times, it has been hypothesized that various risk factors promote Aβ and tau-related pathological changes before the onset of clinical symptoms in AD. One of the formidable challenges of the twenty-first century is to identify these risk factors and enable early detection of pathophysiological alterations at the cellular and biochemical level so that effective treatments can be designed against this devastating disease. A significant risk factor associated with sAD that has received a considerable attention in recent times is Type 2 Diabetes (T2D) (Li et al., 2015a).

### Type 2 diabetes

Diabetes mellitus is a chronic metabolic disorder that is increasing worldwide at an alarming rate. It is estimated that 387 million people are affected by Type1 and T2DM and this number is expected to reach 552 million by 2030 (American Diabetes, 2009). The financial costs for the treatment of diabetes and support for the patients presents a significant healthcare challenge for any country across the world.

The most prevalent subtype of diabetes is the Type 2 diabetes mellitus (T2DM) that comprises 95% of this disease. The salient features of T2DM are high levels of blood glucose (hyperglycaemia), hyper-insulinemia, and insulin resistance (Taylor, 2012). Insulin resistance arises due to decreased insulin sensitivity of muscle, liver, and fat cells to insulin. Another prominent feature of T2DM is the formation of human islet amyloid polypeptide that causes pancreatic β-cell dysfunction (Marzban et al., 2003). Both these features ultimately result in a reduced uptake of circulating blood glucose for glycogenesis eventually leading to chronic hyperglycemia as one of the pathological hallmarks of T2DM.

What is the evidence that there is a patho-physiological link between Diabetes and AD?

### Evidence from epidemiological studies

Epidemiological studies show that T2DM increases the risk of AD by at least 2-fold (Barbagallo and Dominguez, 2014). In a study cohort recruited from Manhattan in 1992–1994 and then in 1999–2001 Cheng et al. demonstrated that T2DM is associated strongly with late-onset AD (LOAD) after adjustment of sex and age. Their findings also suggested that the link between T2DM and LOAD is partly mediated by cerebrovascular pathology (Cheng et al., 2011). Recent studies from Li et al. report that T2DM in an elderly Chinese population with mild cognitive impairment (MCI) influences the progression to AD, while no change is observed in age-matched controls (Li et al., 2016). These data are supported by longitudinal studies conducted by Leibson et al. and Huang et al. in which patients with adult onset diabetes exhibited a significantly higher risk of developing AD than age-matched subjects without T2DM (Leibson et al., 1997; Huang et al., 2014). Epidemiological studies have also examined the association between ApoE4 genotype and diabetes or insulin resistance, although the reports are controversial. For instance, a longitudinal study in Japanese-American men demonstrated that ApoE4 increases the risk of LOAD in individuals with T2DM (Peila et al., 2002). In contrast, population-based studies conducted by Marseglia et al. show an association between T2DM and risk of dementia only in ApoE4 non-carriers (Marseglia et al., 2016).

### Evidence from neuroimaging studies

The evidence from epidemiological studies has recently been corroborated by findings from neuroimaging studies (PET and MRI) that monitor the structural alterations in brains of patients affected by AD and T2DM. The data from the neuroimaging studies reveals a considerable overlap between the vulnerable brain regions in both patient groups.

AD is generally associated with widespread brain atrophy that initiates in the transentorhinal and entorhinal cortex in the early stages and then spreads to the remaining neocortical areas (Fjell et al., 2015). Neuroimaging studies show that the widespread pattern of neurodegeneration caused by AD in the limbic and neocortical regions correlates closely with cognitive deficits and behavioral patterns that AD patients exhibit. However, determining the early stages of AD pathophysiology still remains a challenge. Recently a comprehensive study based on high-resolution MRI on people with MCI and AD revealed that the earliest signs of AD pathology appeared in the cholinergic cells of the nucleus basalis of Meynert (NbM) in the basal forebrain (Schmitz et al., 2016).

Interestingly, neuroimaging studies of brains in individuals with T2DM also show structural alterations that resemble those seen in AD patients. In an elegant study conducted by Moran et al. 350 people with T2DM and 363 control individuals were assessed for cognitive functions with an MRI scan to identify the regional distribution of brain atrophy to identify the causes of cognitive impairment in T2DM patients (Beckett et al., 2015; Moran et al., 2015). The investigators found that T2DM was associated with more cerebral infarcts and reduced volumes of gray matter, white matter, and hippocampus compared to non-diabetic individuals. It was further observed that in people with T2DM, gray matter loss was most prominent in medial temporal, anterior, cingulate, and medial frontal lobes—the regions maximally vulnerable to AD. Moreover, cognitive functions, and in particular visuo-spatial skills, were markedly affected in the T2DM group. Another study by Roberts et al. examined the associations of T2DM with imaging biomarkers and cognitive abilities in 1,437 elderly individuals without dementia (Roberts et al., 2014). They found that midlife T2DM was associated with reduced hippocampal and whole brain volumes strongly indicating decline of cognitive functions later in life. Wennberg et al. conducted a study on 233 cognitively normal individuals who were assessed for fasting blood glucose and cortical thickness measurements by MRI (Wennberg et al., 2016). This study showed that higher blood glucose was associated with reduced average thickness in the AD vulnerable regions. Based on these observations, the authors conclude that the brain atrophy in T2DM, evident from imaging studies, bears striking resemblance to that seen in preclinical AD.

### Shared pathophysiology between AD and T2DM

PET and MRI studies show marked impairment of glucose and energy metabolism in both T2DM and AD (Umegaki, 2014). In addition, amyloidogenesis remains a salient feature in both these diseases. Extracellular β-amyloid plaques form one of the characteristic features of AD. Likewise, deposits of amyloidogenic peptide (IAPP) are detected in the pancreatic islets of Langerhans of T2DM patients (Haataja et al., 2008). Interestingly, diabetic mice overexpressing IAPP develop oligomers and fibrils with more severe diabetic trait similar to AD mouse models that overexpress APP (Marzban et al., 2003). Advanced glycation end products (AGE) and their receptors (RAGE) accumulate in the sites of diabetic complications such as kidney, retina, and atherosclerotic plaques under conditions of ER and oxidative stress (Nowotny et al., 2015). Similarly, glycated products of Aβ and tau form in transgenic AD models as well as in post-mortem brains of AD patients under similar stress conditions and form an important component of neurofibrillary tangles (Schedin-Weiss et al., 2014). Moreover, additional traits of synaptic dysfunction, activation of the inflammatory response pathways and impairment of autophagy are pathological features common to both AD and T2DM (De Felice and Ferreira, 2014; Carvalho et al., 2015).

**Figure 1:**
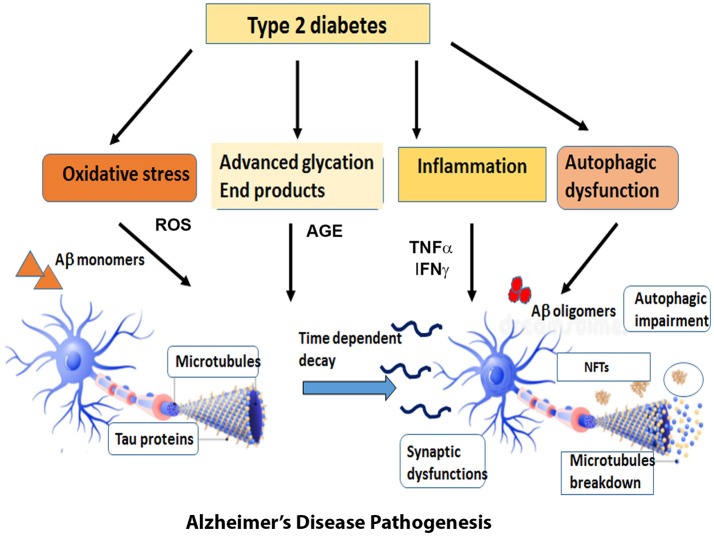
Overview of the diverse mechanisms by which Type 2 Diabetes can cause AD pathogenesis. Type 2 Diabetes accompanied by insulin resistance and hyperglycemia gives rise to metabolic problems in the brain and other target tissues that sets off a cascade of pathogenic processes such as oxidative stress, inflammatory responses, advanced glycation products and autophagic dysfunction. The reactive oxygen species generated by these pathways expedite the process of neuronal death. At the same time, the insulin resistance impairs the downstream signaling pathways and exacerbates the formation of Aβ oligomers and aggregates of hyperphosphorylated tau. The cumulative effect of all these factors expose the neurons to a range of assaults and gradually result in the loss of synapses and neuronal death.

The first section of this review will discuss the impact of brain insulin resistance evident in T2DM on the two hallmarks of AD, Aβ, and tau and describe the possible mechanisms that interconnect AD and T2DM in the areas of synaptic dysfunction, inflammation, and autophagic impairment (Figure 1).

## Alzheimer's disease: insulin resistance in the brain

Brain insulin resistance is a significant, yet often over-looked feature of AD (De Felice et al., 2014). Insulin released from the pancreas is transported to the brain via the blood brain barrier using a receptor-mediated mechanism. While the crucial role of insulin response in the peripheral tissues is well documented, there are very few reports about the function of insulin in the central nervous system. The insulin levels in human and rodent brain tissue are relatively low compared to circulating levels (Talbot, 2014). There are some reports about reduced insulin levels in the AD brains; however, this finding was not significant compared to the age-matched controls (Stanley et al., 2016). Recently several studies have reported reductions of insulin mRNA in AD, however the results of *de novo* insulin synthesis have been controversial (Blázquez et al., 2014). Thus, it is hypothesized that majority of brain insulin comes from the peripheral tissues and the role of insulin produced in the CNS is still unclear.

Although, the brain was initially considered a non-insulin target organ; several studies indicate the widespread distribution of insulin receptors (IR) in the brain particularly in the olfactory bulb, cortex, hippocampus, and hypothalamus indicating an intricate “neuroregulatory” role for insulin (Kim and Feldman, 2015). IRs in the brain are enriched in neurons compared to glia, and concentrated at the synapse. However, contrary to the IRs found in the peripheral tissues, the primary function of brain IRs is not glucose transport and metabolism. Instead, the brain IRs perform diverse functions including homeostatic regulation, modulation of synaptic plasticity, and neurotransmission and age related neurodegeneration (Plum et al., 2005).

The crucial function of glucose uptake and utilization in the brain is carried out by Glucose transporter 4 (GLUT4). Insulin activates GLUT4 gene expression and translocation from the cytosol to the plasma membrane to regulate glucose homeostasis in the brain and maintains energy requirements for a variety neuronal functions (Chiang et al., 2001; Reno et al., 2017).

### Insulin signaling in the brain

A large number of recent studies provide compelling evidence that deficits in insulin signaling, arising due to insulin resistance, occurs in AD (Talbot et al., 2012; Mullins et al., 2017). FDG-PET studies of the brains of “early stage” AD patients have demonstrated reduced glucose uptake leading Suzanne de la Monte and colleagues to classify AD as “Type 3 Diabetes”(de la Monte and Wands, 2008).

**Figure 2:**
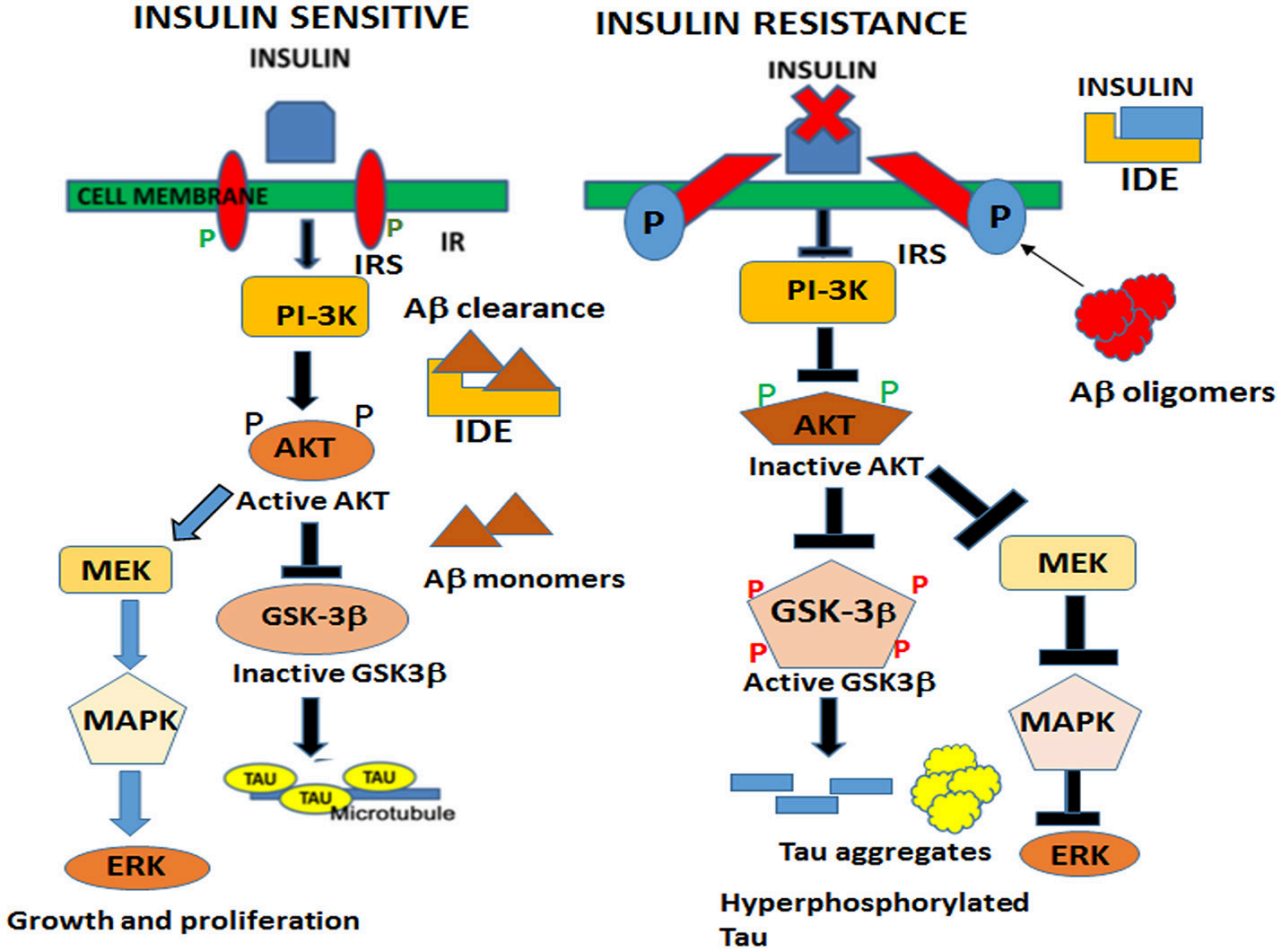
Neuronal signaling mechanisms in a state of insulin sensitivity and insulin resistance. In the insulin sensitive state insulin binds to the receptor and activates the insulin receptor tyrosine kinase that initiates a cascade of phosphorylation events at the IRS/PI3K/AKT and Ras/Raf/ERK pathways. AKT phosphorylates GSK-3β at the inhibitory serine 9 residue and allows tau to maintain its physiological function of binding to microtubules and facilitates normal axonal transport of neuronal vesicles. In a state of insulin resistance, GSK-3β is activated by phosphorylation at Tyrosine 216 residue and hyperphosphorylates tau at pathological epitopes. Hyperphosphorylated tau then detaches from the microtubules and aggregates to form neurofibrillary tangles. Likewise, in the presence of excess insulin, the insulin degrading enzyme (IDE) is unable to degrade and facilitate clearance of Aβ oligomers that act as a competitive substrate for insulin. Thus, insulin resistance facilitates the formation of both Aβ and tau oligomers.

In the brain, insulin and IGF signaling mechanisms are crucial for maintaining synaptic plasticity and cognitive functions (Boucher et al., 2014). Once insulin binds to the IR, it is activated by auto-phosphorylation of several tyrosine residues which in turn activates insulin receptor substrates 1 (IRS-1) and 2 that initiate a host of downstream signaling cascades through phosphatidylinositol-3-kinase (PI3K) (Figure 2). PI3K then activates AKT, which phosphorylates GSK-3β at serine 9 residue thereby inhibiting its activity and resulting in glycogen synthesis (Avila et al., 2012). There are numerous reports that GSK-3β is one of the key tau kinases playing a central role in AD pathogenesis (Chatterjee et al., 2009; Hernandez et al., 2013). Physiologically GSK-3β is involved in maintaining synaptic plasticity and regulates NMDA receptor-mediated long-term potentiation (LTP) and long-term depression (LTD) effects at the synapses (Bradley et al., 2012). In the disease state however, GSK-3β is hyperactive and phosphorylates tau at pathological epitopes. Hyperphosphorylated tau then aggregates to form neurofibrillary tangles (Avila et al., 2010). GSK-3β is also a key mediator of apoptosis suggesting that it might activate neuronal loss in degenerative diseases with increased production of Aβ (Qu et al., 2014). Emerging data also shows that PI3K/AKT pathway regulates synaptic plasticity by stimulating excitatory and inhibitory cell membrane receptors, enhances neurotransmitter activities and increases cortical glucose metabolism in the brain regions that are important for learning and memory (Farrar et al., 2005). In parallel, insulin activates the MAPK pathways leading to Ras activation at the plasma membrane and the sequential activation of Raf, MEK, and ERK (Zhang et al., 2011) (Figure 2). Although a direct role of the components of the MAPK pathway in mediating AD pathology has not yet been deciphered, recent reports indicate that ERK plays a crucial role in synapse formation and learning/memory functions implying that it may have additional neuroprotective functions (Thiels and Klann, 2001). Apart from PI3K/AKT and Ras/Raf/MAPK pathways, less well-defined roles in AD pathogenesis are played by mammalian target of rapamycin (mTOR) and its downstream targets that regulate neuronal survival and nutrient sensing. mTOR regulates protein synthesis by phosphorylating the key substrates of the translational machinery namely the eukaryotic initiation factor 4E-binding protein (4E-BP1) and p70S6 kinase (S6K1). Rapamycin inhibits mTOR *in vivo* and halts cellular growth and proliferation (Showkat et al., 2014).

It is hypothesized that in an insulin-resistant state these downstream signaling pathways are compromised leading to increased levels of Aβ oligomers and hyperphosphorylated tau not only due to a dysregulation of the downstream kinases but also due to an impairment of autophagic clearance that arises as a result of imbalance of the mTOR and autophagy pathways. Autophagic dysfunction, which is an emerging feature of AD causes the progressive accumulation of toxic proteins and eventually leads to neuronal death (Orr and Oddo, 2013).

From the epidemiological and brain neuroimaging studies it is evident that insulin and IGF signaling pathways are important for preservation and maintenance of learning and memory processes that are compromised in AD. Supporting this, intranasal insulin administration improves learning and memory functions in AD patients, emphasizing the shared pathophysiology in both diseases (Benedict et al., 2007).

## Crosstalk of T2DM and Aβ pathology in AD

One of the hallmarks of AD pathology is the formation of extracellular amyloid plaques composed of insoluble deposits of amyloid-β protein aggregates. Aβ is generated from the proteolytic cleavage of amyloid precursor protein (APP) by a sequence of enzymatic reactions from BACE-1, β and γ-secretase complex (Vassar, 2004). It is hypothesized that lower concentration of Aβ remains in the soluble form and subject to clearance after degradation, while higher concentrations aggregate into insoluble plaques that are resistant to degradation (Esparza et al., 2016). While there is no evidence that AD brains secrete more soluble Aβ than normal brains, a recent body of evidence suggests that an increased accumulation of insoluble Aβ plaques arise due to impaired clearance of Aβ protein (Wildsmith et al., 2013).

### Impact of hyperinsulinemia on Aβ

Emerging studies indicate that hyperinsulinemia may confer risk of AD by modulating Aβ toxicity. The enzyme responsible for degrading the Aβ protein is Insulin Degrading Enzyme (IDE), which also degrades insulin (Qiu and Folstein, 2006). In T2DM, peripheral hyperinsulinemia increases the concentration of insulin which acts as a competitive substrate for IDE and inhibits the degradation of Aβ that gradually accumulates to form insoluble plaques. IDE has previously been identified as the primary regulator of Aβ in both neurons and glia (Son et al., 2016). In a recent study Farris et al. demonstrated that homozygous deletions of IDE gene (IDE–/–) in mice, resulted in 50% decrease in Aβ clearance in brain homogenates and primary neuronal cultures (Farris et al., 2003). The IDE depleted mice exhibited increased cerebral accumulation of endogenous Aβ, in addition to the hyperinsulinemia and glucose intolerance that characterize T2DM. Hyperinsulinemia also affects APP transport. *In vitro* studies by Gasparini et al. demonstrated that in βAPP overexpressing N2a cells and primary neuronal cultures, insulin decreases the intracellular accumulation of Aβ by promoting the transport of βAPP/Aβ from the trans-golgi network to the plasma membrane (Gasparini et al., 2001). Thus, in addition to inhibiting the degradation of Aβ by IDE, insulin increases the concentration of extracellular Aβ by modulating the trafficking of APP. The investigators also show the involvement of receptor tyrosine kinase/mitogen-activated protein (MAP) kinase pathway in regulating intracellular Aβ transport (Matos et al., 2008).

### Impact of insulin resistance on Aβ

Studies by Cheignon et al. have shown that inhibition of PI3K leads to reduced Aβ production (Cheignon et al., 2018). When they crossed neuron specific IR knockout mice to APP overexpressing Tg2576 transgenic mice, this study found that loss of insulin signaling in the brain reduced production of Aβ and amyloid plaque deposits. However, the decrease of Aβ burden was not sufficient to rescue the mortality observed in these transgenic mice. Nevertheless, this data provides strong evidence that IRS signaling plays an important role in modulating the Aβ deposition.

Investigating the effect of diet-induced insulin resistance on amyloidosis in Tg2576 AD transgenic mouse models, Ho et al. found that the animals displayed the first signs of memory deficits only at 6 months of age. These animals also maintained normal circulating insulin levels and glucose metabolism till they were 13 months old when there was evidence of plasma hyperinsulinemia. Interestingly, despite high production of Aβ and senile plaques in the brain, these mice showed no evidence of neuronal death and neurofibrillary tangles. However when these mice were reared on a high fat diet they developed non-insulin dependent diabetes mellitus, that led to increased production of Aβ40 and Aβ42, higher activation of γ-secretase as well as GSK-3α and GSK-3β. Biochemical evidence has shown that these mice displayed lower PI3K and AKT activation signals denoting insulin resistance with impaired learning and memory functions (Ho et al., 2004; Chouliaras et al., 2010).

### Impact of hyperglycemia on Aβ

Elevated plasma glucose levels are a common pathological feature of T2DM affected individuals. A compelling link between glucose metabolism and AD was established in a study conducted in transgenic APP/PS1 murine models (Macauley et al., 2015). The researchers observed that the induction of acute hyperglycaemia in young mice increased the production of Aβ and lactate in hippocampal interstitial fluid and this was associated with an increase in neuronal activity (Minkeviciene et al., 2009). These effects worsened in aged AD mice with increased Aβ plaque pathology. These findings suggest that transient hyperglycemia associated with T2DM can initiate the formation of Aβ plaques. In this study, the authors also show that hippocampal ATP sensitive potassium (KATP) channels act as metabolic sensors of the alterations in glucose concentration with changes in electrical activity and extracellular Aβ deposition.

In addition, aberrant glucose metabolism activates glycation reactions that leads to the formation of advanced glycated end products (AGE). Elevated AGE levels in the circulation and in the brain have been associated with cognitive impairments in AD patients (Li et al., 2013). Numerous studies show that there is an increased accumulation of AGE in the brain of diabetic rats implying that AGE products impair the removal of Aβ42 and induce Aβ aggregation in the brain (Moreira et al., 2003).

AGEs enhance the expression of its receptor RAGE, which is also a presumed receptor for Aβ (Srikanth et al., 2011). Recent reports have shown increased RAGE expression in the astrocytes and microglia of AD brains (Solito and Sastre, 2012). Also, studies on triple transgenic model of AD (3xTg AD) expressing 3 dementia-related transgenes, namely APPSWE, PS1M146V, and tauP301L and raised on high-fat diet as well as STZ-induced diabetic APP/PS1 dual transgenic AD mice demonstrate increased RAGE expression in neurons and astrocytes with an activation of pro-inflammatory cytokines and enhanced decline of cognitive and memory functions (Choi et al., 2014). As a consequence of the damaging inflammatory responses, RAGE causes vascular complications in AD and T2DM. Neuron-specific overexpression of dominant negative RAGE results in restoration of cognitive functions and stops the progression of neuropathological changes in AD mice (Liu L. P. et al., 2009). Treatment of transgenic AD mice with RAGE inhibitor decreases microglial activation and Aβ production (Criscuolo et al., 2017). Interestingly, soluble form of RAGE (sRAGE) is neuroprotective (Deane et al., 2012; Lee and Park, 2013).

O-GlcNAcylation is a nucleocytoplasmic post-translational modification that occurs abundantly in neurons and protects against Aβ-mediated neuronal toxicity. Recent studies show that a moderate level of O-GlcNAcylation is neuroprotective and decreases formation of Aβ production by inhibiting γ-secretase activity (Hanover and Wang, 2013). Insulin resistance and hyperglycemia increases the level of O-GlcNAcylation but the amelioration of γ-secretase activity is counterbalanced by the accumulation of glycated end products that are ultimately toxic to neurons.

### Impact of dyslipidemia on Aβ

Insulin plays a crucial role in lipid metabolism and impairments in insulin signaling lead to increased lipolysis and elevated synthesis of free fatty acids (FFA) (Wilson and Binder, 1997). Human brain produces approximately 30% of total body cholesterol, hence slight alterations in lipid metabolism can have profound effects on cognitive function. Recent studies show that the interaction between cholesterol and APP in the plasma membrane is necessary for Aβ synthesis and clearance. Tg2576 AD mice raised on a diet supplemented with high fat and high cholesterol displayed increased production of Aβ. When these animals were treated with cholesterol lowering drugs the brain amyloid levels were reduced by more than 2-fold (Nizari et al., 2016).

### Impact of Aβ oligomers on insulin resistance

Soluble oligomers of Aβ42 have been shown to be the most toxic species to the neurons. In the brains of AD patients with MCI, Aβ oligomers have been shown to correlate with rapid cognitive decline (Ferreira et al., 2015). Also, in the brain and CSF of AD patients the level of soluble oligomers ranging from trimer to 24-mer or higher is significantly elevated when compared to levels found in non-demented controls (Jimenez et al., 2014). Studies in human APP overexpressing Tg2576 AD mice show that the onset of memory deficits correlate with production of soluble Aβ oligomers. Small oligomers of Aβ, particularly dimers and trimers that are formed within the neurons *in vitro* are secreted into conditioned medium. The treatment of rat hippocampal slices or live animals with these oligomers showed potent LTP defects and cognitive deficits (Puzzo et al., 2017). Similarly, synaptotoxicity is observed when soluble Aβ oligomers isolated from the brains and CSF of AD patients are applied to brain tissue slices or live animals (Minkeviciene et al., 2009). These, inhibitory effects are blocked by Aβ antibodies and γ-secretase inhibitors (Goure et al., 2014). This LTP blockage is also restored by treatment with 1μM insulin (Sakono and Zako, 2010). These observations were confirmed by other investigators who demonstrated that Aβ42 oligomers were more potent in blocking LTP than monomers and this effect was rescued by insulin by preventing Aβ oligomerization (Selkoe, 2008; Mucke and Selkoe, 2012).

Recent evidence indicates an intimate connection between brain insulin resistance and the formation of Aβ oligomers. In a study by Zhao et al. the scientists found that the treatment of primary hippocampal neurons by soluble Aβ oligomers results in a profound loss of insulin receptors from the neuronal surface. In addition, Aβ oligomers were found to increase the AKT phosphorylation at Serine-473 residue which results in insulin resistance (Zhao and Townsend, 2009). In addition, Aβ oligomers inhibit insulin signaling by phosphorylating IRS1 at inhibitory serine residues via the JNK/TNF alpha pathway (De Felice et al., 2009). Conversely, treating AD patients and transgenic animals with intranasal insulin lowers the concentration of soluble Aβ and improves memory (De Felice et al., 2009). Aβ-induced insulin resistance was also observed in the Familial AD (5XFAD) transgenic mouse models that overexpress high levels of mutant APP and PS1 and display severe amyloid pathology since 2 months of age (Mosconi et al., 2008).

Overall these studies demonstrate a strong impact of insulin resistance, hyperglycemia, dyslipidemia, and other hallmarks of T2DM on the pathological effects of Aβ amyloidogenesis as observed in AD.

## Crosstalk of T2DM and tau pathology in AD

The traditional physiological function of tau protein is to promote assembly and stabilization of microtubules, though newer, atypical functions are now being reported (Sotiropoulos et al., 2017). There are about 80 Ser/Thr and 5 Tyr phosphorylation sites on tau that are phosphorylated by the key tau kinases namely GSK-3β, MARK/PAR1, and CDK5 (Stoothoff and Johnson, 2005; Chatterjee et al., 2009). While in the normal brain, tau contains 2–3 moles of phosphate/mole of tau protein; in abnormal situations as seen in AD brains and other tauopathies, tau becomes hyperphosphorylated with 6–9 moles of phosphate/mole of tau and aggregates to form intracellular neurofibrillary tangles, one of the pathological hallmarks of AD (Iqbal et al., 2010).

A wealth of emerging studies in recent years indicate that the density of the neurofibrillary tau tangles deposited at the neocortex of the brains of MCI patients correlated more strongly with the severity of the disease (Bierer et al., 1995; Nelson et al., 2012). This study showed that learning and memory defects were more acute in patients with higher accumulation of the tangles in the temporal lobe which is the brain region associated with learning and memory. Thus, the post translational modifications of tau (hyperphosphorylation, truncation, acetylation, and glycation) and associated cellular and biochemical changes that cause these abnormal structural alterations are of potential interest for the development of tau targeted therapeutic studies.

Two of the important features of T2DM namely insulin resistance and hyperglycemia are able to influence post translational modifications of tau and exacerbate tau pathology, discussed below:

### Impact of insulin resistance on tau kinases and phosphatases

Under physiological conditions, a host of kinases and phosphatases regulate the intricate balance between tau phosphorylation and dephosphorylation to maintain neuronal homeostasis. Several protein kinases such as GSK-3β, CDK5, MARK, PKA, PKB/AKT, and MAPK including ERK1/2, c-JUN N terminal kinase (JNK) and p38 are the important kinases that phosphorylate tau (Avila, 2008; Gómez-Sintes et al., 2011).

Among these kinases, AKT and GSK-3β are located downstream of one arm of the insulin signaling pathway while components of the MAPK pathway lie downstream of the other arm (Fröjdö et al., 2009). AKT phosphorylates GSK-3β at the inhibitory Serine-9 residue and maintains GSK-3β in the inactive form. However, under conditions of insulin resistance, GSK-3β is converted to its active form by phosphorylation at Tyrosine-216 residue. Active GSK-3β then hyperphosphorylates tau to generate the pathological epitopes AT8, AT100, and PHF1 which make up the pre-tangles and NFTs in the AD brains (Clodfelder-Miller et al., 2005).

Several other studies have found that p70S6 ribosomal protein kinase—an AKT/mTOR substrate can directly phosphorylate tau and upregulate its synthesis through phosphorylation of S6 (Yoon, 2017).

These key pathways, namely PI3K/AKT, MAPK, and mTOR/p70S6K are regulated by insulin binding to the insulin receptor and trigger downstream phosphorylation events (Fröjdö et al., 2009). Thus, it is not surprising that in an insulin resistant state, insulin is unable to activate these pathways thereby disrupting physiological tau phosphorylation. Additionally, in diabetic brains p38 and JNK activation can cause insulin resistance by inhibiting the insulin receptor substrate and trigger tau hyperphosphorylation and pathological events (Wu et al., 2013).

Tau is also regulated by phosphatases especially PP2A that dephosphorylates it at crucial residues Thr205, Thr212, and Ser 262 that are phospho-epitopes of GSK-3β and MARK/PAR-1. In addition, PP2A dephosphorylates the kinases GSK-3β and p70S6K to maintain tau phosphorylation at a physiological level (Gratuze et al., 2017). Interestingly, several researchers have shown a downregulation of PP2A in both T1DM and T2DM mice suggesting that insulin resistance might exacerbate tau phosphorylation by downregulating PP2A (Qu et al., 2011; Jung et al., 2013).

The impact of isolated CNS-specific insulin resistance on tau phosphorylation was investigated *in vivo* by Schubert et al. using NIRKO mice where the brain/neuron specific IR gene was conditionally inactivated. They found that NIRKO mice displayed a complete loss of insulin-mediated PI3K/AKT signaling resulting in reduced phosphorylation of GSK-3β at Serine-9 and increased tau phosphorylation. However, these animals did not exhibit any change in survival or learning and memory defects or basal brain glucose metabolism (Schubert et al., 2004). In another study modeling peripheral insulin resistance in IRS2 knock out (KO) mice Schubert and colleagues show that neurofibrillary tangles composed of hyperphosphorylated tau aggregates accumulate in the hippocampus of IRS2 KO mice, revealing a direct molecular link between diabetes and Alzheimer's disease (Schubert et al., 2003). Likewise, Cheng et al. in IGF KO mouse brains displayed increased tau phosphorylation at Ser-396 and Ser-202 residues both of which are implicated in neurodegeneration (Cheng et al., 2005). In STZ-induced Type 1 diabetic mouse models, Planel and colleagues observed a robust increase in tau hyperphosphorylation that prevented tau from binding to microtubules (Planel et al., 2007). The scientists further observed that a downregulation in the activity of PP2A in these transgenic models exacerbated tau pathology. Similarly, Yazi and colleagues administered STZ to induce diabetes in pR5 mice expressing P301L mutation. Compared to non-transgenic controls, the pR5 mice displayed massive tau hyper-phosphorylation with the formation of neurofibrillary tangles by 6 months of age (Ke et al., 2009). In addition, other studies show that treatment of STZ-induced diabetic mice with GSK-3β inhibitors improves learning and memory functions (King et al., 2013). All these studies study provide compelling evidence that both Type 1 and Type 2 diabetes can accelerate onset and disease progression in individuals with a predisposition to developing tau pathology.

### Impact of insulin resistance on tau cleavage

In addition to hyperphosphorylation, another abnormal post-translational modification of tau is truncation. Tau is cleaved by a host of proteolytic enzymes including caspases, calpains, thrombins, and puromycin-sensitive amino peptidase. These truncated tau fragments lack both N terminal and C terminal fragments and form the core component of NFTs (Karsten et al., 2006; Zilka et al., 2006).

In AD brains, caspases are activated causing tau protein to be cleaved at several residues. The C-terminal cleavage of tau by caspase-3 gives rise to Asp421 residue that has a higher propensity of aggregation and is found to be associated with neurofibrillary pathology in AD brains. The presence of Asp421 truncated tau in the neurofibrillary aggregates observed in the neurons of double transgenic mice (Tet/GSK-3β/VLW) and in P301S mouse models of tauopathy indicates that the formation of Asp421 cleavage product is an important step toward formation and maturation of tau aggregates (Basurto-Islas et al., 2008; Gendron and Petrucelli, 2009; Gómez-Sintes et al., 2011).

Diabetes has been known to stimulate apoptosis by the activation of caspase-3 in affected tissues (Savu et al., 2013). Using an animal model of T2DM, Kim et al. in their studies demonstrated an increased level of tau phosphorylation and cleavage in the brains of db/db mice which are models for diabetic dyslipidemia (Kim B. et al., 2013). Feldman and colleagues found that hyperglycemia promotes tau cleavage by activation of caspases (Kim et al., 2009). Thus, these studies demonstrate that T2DM enhances the formation of tau truncated fragments by caspase activation that contribute toward an increased risk of AD in diabetic patients.

### Impact of hyperglycemia on other posttranslational modifications of tau

Acetylation is a post translational modification in which the acetyl group from acetyl CoA is reversibly transferred to lysine ε amino group in the tau protein. This process is modulated by acetyltransferases and deacetylases (Cook et al., 2014). Cohen et al. observed that tau acetylation at the key lysine residues at K/163/280/281/369 was crucial for tau-microtubule binding interactions and microtubule stabilization. Using cellular and transgenic mouse models as well as human brains from a wide spectrum of tauopathies this group has demonstrated that tau acetylation disrupts the tau-microtubule binding interactions and promotes pathological tau aggregation. This group further demonstrated that acetylated tau was associated with the formation of insoluble tau NFTs in tau transgenic mice and human tauopathies indicating “acetylation” as a pathogenic post-translational modification of tau (Cohen et al., 2011).

Protein acetylation plays an important role in intermediary metabolism and metabolic disorders including T2DM. Mass spectrometry on STZ-induced diabetic rats showed high levels of lysine-acetylated proteins in their kidney cells compared to control animals (Kosanam et al., 2014). Also, treatment of murine aorta cells with high glucose or FFA to induce short term diabetes causes increased levels of lysine acetylation (Samuel et al., 2017). Although, there are very few reports, aberrant acetylation of tau in T2DM may interfere with the physiological functions of microtubule binding and assembly predisposing cytoplasmic tau toward the formation of aggregates (Irwin et al., 2013; Trzeciakiewicz et al., 2017).

Glycosylation involves the attachment of oligosachharide moieties to proteins and lipids. O-glycosylation is the linkage of sugar residues to the hydroxyl groups of Serine or Threonine residues while N-glycosylation involves attaching the sugar moieties to the amine group of the aspargine residues in proteins (Wang et al., 1996). Impaired OGlcNAc cycling is implicated in AD. In *post-mortem* AD brains Zhu et al. have demonstrated a significant decrease of O-GlcNAc glycosylation of tau proteins compared to controls (Zhu et al., 2014). Other studies from AD patients show hyperphosphorylated but hypo-O-GlcNAc glycosylated tau implying that phosphorylation and O-glycosylation at Serine and Threonine residues act in opposition (Wang et al., 1996; Iqbal et al., 2010). *In vitro* studies in NMR and Mass spectrometry analysis by Smet-Nocca et al. demonstrated that tau hyper phosphorylation at residues Ser-396 and Ser-404 were reduced by O-glycosylation at Ser-400 (Smet-Nocca et al., 2011). Treatment of mouse models of AD with OGA inhibitor Thiamet-G (that increases the levels of O-GlcNAcylation of tau) was found to decrease the levels of NFT burden and pathological tau species and slow down disease progression (Robertson et al., 2004; Gong et al., 2005; Yuzwa et al., 2012). However, it is interesting that Thiamet-G decreases tau phosphorylation over a short period of time and prolonged OGA inhibition has no effect on phosphorylation. This is probably due to cellular adaptability over time. Compared to O-glycosylation, *in vitro* studies show that N-glycosylated tau isolated from AD brain promotes tau hyperphosphorylaton (Liu Y. et al., 2009). Taken together these studies demonstrate that glycosylation exerts varying effects on tau hyperphosphorylation.

Although it is still speculative, it has been hypothesized that abnormal glucose metabolism in the brain induced by T2DM may lead to decreased brain O-GlcNAc levels. This reflects a failure in the neuroprotective mechanism in the brain and triggers the cascade of tau pathology enabling disease progression.

### Impact of tau on insulin resistance

While insulin resistance stimulates tau hyperphosphorylation and aggregation, in a recent study Rodriguez-Rodriguez and colleagues have shown that pathological alterations in tau (hyperphosphorylation and aggregation) accumulates insulin. In this study, the researchers have shown that insulin accumulates in the sarcosyl-insoluble fractions of the AD brain. Moreover, the researchers found increased accumulation of insulin *in vivo* in the brains of tau overexpressing P301S mice and SHSY5Y cells as well as in okadoic acid treated primary neuronal cultures. Both the cells and primary neurons demonstrated increased insulin uptake from the surroundings that eventually led to insulin resistance (Rodriguez-Rodriguez et al., 2017). Another study shows impaired insulin sensitivity in hippocampal slices from tau KO mice compared to litter-mate controls (Marciniak et al., 2017).

These studies suggest a complex relationship between tau and insulin resistance as it is evident that not only insulin resistance can exacerbate tau pathology but pathological tau phosphorylation or absence of tau can affect neuronal insulin resistance.

## Synaptic dysfunctions at the crossroads of AD and T2DM

There is widespread neuronal loss and atrophy of the cortex and hippocampus in the brains of AD patients (Sheng et al., 2012). The cognitive failure in AD patients is accompanied by loss in synapses and neuronal cell death with a marked reduction in brain volumes particularly at the entorhinal cortex and hippocampus. Although plaques and tangles characterize the neuropathological features of AD, the closest correlation to the severity of disease progression is the synapse loss that occurs in the disease. There are conflicting reports as to whether the amyloid plaques or NFTs correlate more strongly with disease progression. Some scientists have reported that the spatiotemporal signature of NFTs of tau correlate more severely with the disease pathology (Nelson et al., 2012; Horvath et al., 2013). However, investigating the “neurodegenerative triad” Tackenberg et al. have shown that the loss of dendritic spines and LTP that occurs early in the disease is mediated by Aβ while the late stage cell death mediated by NMDAR requires tau phosphorylation (Tackenberg and Brandt, 2009).

Alterations in synaptic transmission and plasticity are also observed in the hippocampus of diabetic animal models including depletion of synaptic vesicles at presynaptic sites and changes of AMPA and NMDA receptors at the postsynaptic sites. Moreover, diabetes affected the synthesis and release of both inhibitory and excitatory neurotransmitters (Gaspar et al., 2016). All of these factors have the potential to activate synaptic dysfunction and widespread neuronal loss thereby predisposing the diabetic brain to AD. In this section the impact of T2DM on Aβ and tau mediated dysfunctions will be elaborated.

### Synaptic dysfunctions in AD

Recent studies indicate that cognitive ability in AD patients is closely related to the alterations of the density of the presynaptic glutamatergic boutons with an elevation of the glutamatergic synapses in MCI patients and a gradual depletion of the boutons with the progression of AD (Bell et al., 2007).

#### Aβ and synaptic dysfunction

There are numerous reports that show Aβ oligomers can cause synaptic dysfunction and toxicity (LaFerla and Oddo, 2005; Shankar and Walsh, 2009). Under physiological conditions, low concentration of monomeric Aβ is essential for maintaining synaptic plasticity and neuronal survival with the improvement of cognitive abilities. Conversely, in the disease state higher concentration of Aβ together with aging causes synaptic dysfunction followed by neuronal loss as seen in AD. Recent studies show that APP and BACE1 KO mice display pronounced defects in LTP and memory functions (Tyan et al., 2012). Puzzo et al. showed that the treatment of mouse brain hippocampal slices with low concentrations (200 picomoles) of synthetic Aβ42 monomers and oligomers increased the LTP. This study suggests that LTP is mediated by α7-nicotinic acetylcholine receptors indicating a presynaptic role of Aβ. Likewise, treatment with nanomolar concentration of Aβ produced synaptic depression. This indicates that an optimal level of Aβ is needed for synaptic transmission (Puzzo et al., 2017).

Elevated levels of Aβ impairs glutamatergic transmission by decreasing the number of AMPA and NMDA receptors on the surface of the neurons. Moreover, increased concentration of Aβ results in the internalization of NMDA receptors enhancing LTD at the synapses. LTD causes a significant loss of dendritic spines that is associated with early symptoms of AD and disease progression (Palop and Mucke, 2010). EEG recording from cortical and hippocampal networks In human APP overexpressing mouse models shows that high levels of Aβ oligomers elicits epileptic and nonconvulsive seizures (LaFerla and Oddo, 2005). Consistent with these findings, *in vivo* calcium imaging of cortical circuits shows that double transgenic (hAPP/PS1) mice have a greater proportion of hyperactive and hypoactive neurons than nontransgenic control (Palop and Mucke, 2010; Puzzo et al., 2017). AD transgenic mice showed an increased neuronal activity in the hippocampal regions before the formation of insoluble amyloid plaques. On treatment with a γ-secretase inhibitor, soluble Aβ levels were reduced and the neuronal activity decreased implying that this effect was a soluble Aβ-dependent effect (Busche et al., 2012). Although the mechanisms are not completely elucidated, these studies suggest that Aβ-dependent effects on synaptic plasticity and neurotransmission are tightly controlled by the activation of α7-nicotinic acetylcholine receptors or NMDARs and involves downstream effector components including p38 MAPK and GSK-3β (Baglietto-Vargas et al., 2016).

T2DM may trigger Aβ-mediated synaptic dysfunction in multiple ways. Hyperinsulinemia in T2DM promotes the formation of Aβ oligomers that cause synaptotoxicity. Investigators have shown that when Aβ oligomers are applied *in vitro* to rat hippocampal slices or *in vivo* to live animals they blocked LTP and inhibited memory formation (Busche et al., 2012). Interestingly, this effect is overcome by 1μm insulin. A similar result was reported by Li et al. who observed that Aβ monomers were more effective than Aβ oligomers in inhibiting LTP and that insulin prevented Aβ-induced LTP defects by blocking Aβ oligomerization (Balducci et al., 2010; Li et al., 2011). Previous studies have shown that Aβ oligomers are capable of binding to insulin receptors which causes an impairment of receptor functions (Bradley et al., 2012; Uranga et al., 2013). In addition, it has been shown that Aβ oligomers alter GSK-3β phosphorylation state and directly impacts the ERK pathway (Magrane et al., 2006; Reddy, 2013).

#### Tau and synaptic dysfunction

Recent research suggests an emerging role of tau at the synapse (Pooler et al., 2014). Physiologically tau is localized primarily in the axons where it binds and regulates microtubule dynamics in a phosphorylation dependent manner (Janning et al., 2014). However, recent isolation and analysis of AD brain-derived synaptoneurosomes indicate that tau is present in both pre-synaptic and post-synaptic compartments (Tai et al., 2012). There are multiple mechanisms by which tau could mediate synaptic function and neuronal excitability. Recent study has shown that tau binds to the post-synaptic protein complex which includes the PSD-95 through Fyn kinase. The interaction of tau and Fyn appears to be crucial for directing Fyn to the postsynaptic compartments where it can regulate the NMDA receptor by phosphorylating one of its subunits. Abnormal tau phosphorylation can disrupt the tau-Fyn interaction and affect postsynaptic receptor targeting (Haass and Mandelkow, 2010). Thus, neurons from conditionally overexpressing P301L tau mice display impaired targeting of excitatory glutamate receptors to dendritic spines. In addition, biochemical analysis of the synaptosomes from these mice display a marked decrease in the levels of synaptic markers (PSD95, Synapsin, NMDAR1, and GluR1) implying that the loss of functional synapses plays an important role in maintaining postsynaptic integrity (Katsuse et al., 2006; Spires-Jones and Hyman, 2014). Electron microscopy of NFT-carrying motor neurons in P301L mice revealed a significant decrease in the number and size of synaptic boutons compared to nontransgenic controls. These studies point out that loss of synapses occur during neurodegeneration. Evidence for tau involvement in regulating neuronal excitability comes from a study in which a reduction in tau levels reduced hyperexcitability in a mouse model of seizure (Holth et al., 2013; Guerrero-Muñoz et al., 2015). Other studies have found that neurons isolated from transgenic Tg4510 mice overexpressing 0N4R P301L mutation were more excitable than neurons from nontransgenic mice (Kopeikina et al., 2013). However, it is worth noting that the animal models mostly express mutant tau protein and therefore could be more representative of FTDP-17 cases than AD.

Although there are fewer reports, glucotoxicity in T1 and T2DM are capable of influencing tau-mediated synaptic impairments. Investigators have shown synaptic defects and cognitive impairments in STZ-induced diabetic models, where genetically ablating tau ameliorated cognitive defects. (Abbondante et al., 2014). Using a mouse model of tauopathy, scientists have observed that under glucose-deprived conditions as observed in hypometabolic AD brains, transgenic mice had impaired memory and reduced LTP accompanied by tau hyperphosphorylation and apoptosis (Lauretti et al., 2017).

### Synaptic dysfunctions in diabetes that influence neurodegeneration

Other than directly influencing Aβ and tau-mediated synaptic defects, diabetes also affects the synthesis and release of key neurotransmitters that may underlie cognitive defects. For instance under chronic hyperglycemia extracellular brain levels of GABA and glutamate were decreased in STZ-induced diabetic animal models (van Bussel et al., 2016). An imbalance between excitatory and inhibitory neurotransmission impaired the cognitive deficits observed in these animals. Diabetes also affects acetylcholine esterase that plays a crucial role in cognitive processes. Recent studies have shown a reduction of cholinergic transmission in the hippocampus of STZ-induced diabetic animals (Molina et al., 2014). Moreover, in the STZ-induced animals, treatment of hippocampal slices with insulin resulted in a significant decrease in the number of NMDA receptors that consequently affected LTP and decreased postsynaptic densities (van der Heide et al., 2005).

Taken together these results suggest that both Type 1 and Type 2 diabetes are able to directly influence Aβ and tau-mediated synaptic dysfunctions. In addition, both these subtypes cause an imbalance of neurotransmitter release and alterations in synaptic plasticity that ultimately leads to memory impairments. Hence, synaptic dysfunctions form a shared pathological trait between AD and T2DM.

## Inflammation: shared pathophysiology of AD and T2DM

Emerging evidence in recent times points toward a compelling link between inflammation and the pathogenesis of Alzheimer's disease since Aβ plaques and NFTs colocalize with glial cells (Serrano-Pozo et al., 2011). T2DM disease pathogenesis in particular involves high levels of ER and oxidative stress response that might trigger the inflammatory cascade (Back and Kaufman, 2012). Additionally, misfolded toxic protein species detected in AD and T2DM may generate oxidative stress and activate inflammatory pathways. In this section, we discuss the role of inflammation in AD and T2DM and how this impacts the shared pathophysiology in both these diseases.

### Inflammation and AD pathology

Recently preclinical, genetic, and bioinformatics studies have shown that the immune system activation accompanies AD pathology. The genome wide association studies (GWAS) between AD and rare mutations in the genes encoding triggering receptor expressed on myeloid cells 2 (TREM2) and myeloid cell surface antigen CD33 provide clear evidence that there is a strong linkage between alterations in the immune system and the progression of AD pathology (Griciuc et al., 2013; Ulrich et al., 2017).

Based on the amyloid cascade hypothesis, the Aβ deposition is followed by immune system activation mediated by glial cells such as microglia and astrocytes. This is supported by electron microscopy studies that show increased accumulation of glial cells surrounding the amyloid plaque deposits in AD brain (Wyss-Coray et al., 2003). However, recent data of cerebrospinal fluid analysis from patients with symptoms of MCI has demonstrated a marked alteration in the inflammatory markers implying their involvement early in the disease pathway (Zotova et al., 2010; Wyss-Coray, 2012). In another significant study, scientists showed that systemic immune challenge elicited by injecting viral mimics of polyriboinosinic-polyribocytidilic acid resulted in “sporadic AD” like features in wild-type mouse models accompanied by Aβ deposition, tau pathology, microglia activation, and reactive gliosis implying that alterations in the immune system can precede AD pathology and drive the disease itself (Michalovicz et al., 2015). In addition, tissue microarrays from patients with neurodegenerative diseases including AD revealed an upregulation of inflammatory components further suggesting an intimate linkage between inflammatory markers and AD, early in the pathogenic cascade (Sekar et al., 2015).

#### Aβ and inflammation

In AD brains, microglia and astrocytes have been known to accumulate around neuritic plaques and are associated with the tissue damages that occur in AD. Aβ oligomers and fibrils are capable of binding to receptors expressed by microglia including CD14, CD36, CD47, a6β1 integrin, RAGE, and Toll-like receptors (TLRs) (Doens and Fernandez, 2014). *In vitro* studies have shown that binding of Aβ to RAGE receptors, helps guide microglia to Aβ deposits and this effect is inhibited by anti-RAGE antibodies (Wyss-Coray, 2012). Binding of Aβ to CD36 or TLR4, on the other hand, results in the production of inflammatory chemokines and cytokines that eventually lead to increased neuronal damage in vulnerable regions of the AD brain (Doens and Fernandez, 2014). Besides the secretion of inflammatory cytokines, microglia are found to phagocytose soluble Aβ oligomers via the extracellular proteases such as neprilysin and insulin-degrading enzyme (IDE). However, there are evidences of Aβ dependent impairment of microglial phagocytosis functions in AD mouse models (Koenigsknecht and Landreth, 2004). Recent studies have shown that microglia isolated from AD transgenic mouse models displayed a substantial reduction in the levels of Aβ-binding scavenger receptor and Aβ-degrading enzyme (Zhao et al., 2017). Interestingly, it has been shown that transient depletion of dysfunctional microglia has no impact on Aβ deposition in an animal model of AD (Morimoto et al., 2011). This is because microglial impairment maybe compensated by inflammatory cytokines such as TNF, IL-1, IL-12, and IL-23 suggesting a negative feedback loop, that might exacerbate the AD pathology (Rubio-Perez and Morillas-Ruiz, 2012). In addition, an upregulation in the levels of inflammatory markers has been demonstrated in animal models of AD or in the brains or CSF of AD patients (Moro et al., 2018).

Recently a huge repertoire of GWAS studies show that structural variants of genes encoding immune receptors TREM2, CD33, and CR1, all of which are expressed in the microglia confer higher risk of AD (Tosto and Reitz, 2013). However, the function of TREM2 deficiency in the progression of AD has been controversial. For instance, while TREM2 deficiency in APP/PS1 mice ameliorated hippocampal Aβ accumulation; the 5XFAD mice displayed an opposite result. In these mice the Aβ pathology was found to develop slower than APP/PS1 mice and increased accumulation of hippocampal Aβ was observed in the absence of TREM2 (Bemiller et al., 2017; Jay et al., 2017). Elevated levels of soluble TREM2 was detected in the CSF of early AD patients suggesting a change in microglial activation in response to neuronal death. Although the exact mechanism still remains to be deciphered, these findings show that impaired TREM2 function plays a vital role in Aβ-mediated AD pathogenesis.

The transmembrane protein CD33 is another microglial receptor the structural variants of which has led to increased risk of AD. A significant study from *post mortem* AD brains has shown the upregulation of CD33 compared to age-matched controls (Jiang et al., 2014). Conversely, the expression of a CD33 variant, namely the protective CD33 (SNP) rs3865444 was downregulated in AD brains and reduced insoluble Aβ deposits (Hu et al., 2014; Li et al., 2015b).

Astrocytes too respond to AD pathogenic stimuli by reactive gliosis. In transgenic AD mouse models exhibiting cerebral amyloidosis the activation of astrocytes occur in the early stages of pathogenesis. In these transgenic animals the astrocytes underwent severe atrophy which preceded the Aβ plaque mediated gliosis (Verkhratsky et al., 2010). Conversely, reducing astrocytes in a transgenic Aβ overexpressing mouse model ameliorated AD pathology (Garwood et al., 2017). The involvement of astrocytes in neuroinflammation entails increased production of cytokines that either affect the neurons directly or via microglial activation (Van Eldik et al., 2016). For instance, NFκβ-mediated activation of astrocytes release the complement protein C3 that can bind to neuronal C3aR and trigger neuronal damage. Another astrocyte signaling molecule is the soluble CD40 ligand that binds to microglial cell surface receptor. This binding interaction releases pro-inflammatory tumor necrosis alpha (TNF-α) that has been widely reported to contribute to tissue damage in AD (Van Eldik et al., 2016). Astrocytes also play a neuroprotective role. Recent studies have also shown that reactive astrocytes surrounding the Aβ plaques take up and degrade Aβ. For instance, in Tg2576 transgenic mice this has been shown to be linked to insulin degrading enzyme (IDE) which plays an important role in Aβ degradation. Reportedly, Aβ exposure of IDE enhanced the number of activated astrocytes surrounding the neuritic plaques (Wyss-Coray, 2012). Other studies have shown that treatment of astrocytes with *ex-vivo* Aβ extracts, increase the secretion of Aβ degrading enzymes (Wyss-Coray et al., 2003). Thus, the Aβ pathogenesis in AD may result in alterations of normal astrocyte functions which may be then trigger downstream inflammatory cascades prompting further neuronal damage in AD.

#### Tau and inflammation

Although there are fewer reports, *in vitro* studies have shown that microglial cells stimulated by Aβ or LPS release pro-inflammatory cytokines such as interleukin-1β and activate tau phosphorylation at the pathological phospho-epitopes via MAPK pathway. This was confirmed by *in vivo* studies in 3xTg mouse model that displays both Aβ and tau pathologies. When these animals were subjected to high dose of LPS treatment, tau hyperphosphorylation was triggered at the pathological epitopes mediated by GSK-3β, CDK5, JNK, and MAP kinases (Barron et al., 2017). Several other studies have shown that the activation of the key kinases CDK5 and GSK-3β by themselves resulted in microglial activation and secretion of IL-1β implying a close link between tau hyperphosphorylation and pro-inflammatory markers. The gene expression profile analysis in a mouse model of tauopathy (rTg4510) which expresses the P301L mutation, revealed upregulation of pro-inflammatory markers such as complement 4B, glial fibrillary acidic protein (GFAP) and osteopontin (Spp1) on treatment with LPS. This result confirms that neuroinflammation modulates tau pathology in the absence of Aβ plaques (Wes et al., 2014). In addition, TREM2 levels were decreased in these transgenic mouse models indicating an alteration in microglial functions (Maphis et al., 2015). In this landmark study, Bhaskar et al. showed that LPS treatment of hTau mice expressing all 6 non-mutated tau isoforms enhanced microglial activation and accelerated tau pathology. These mice were deficient in microglia-specific fractalkine receptor (CX3CR1) that caused an exacerbation of tau hyperphosphorylation via p38/MAPK pathway. Further, studies in a P301S mutant human tau transgenic mice demonstrated that these animals displayed synaptic pathology and microgliosis before the onset of tangle formation confirming that microglial activation occurs early in the disease pathway. In this study, the researchers have shown that the immunosuppression of young P301S transgenic mice by FK506 significantly diminished tau pathology and increased their lifespan (Yoshiyama et al., 2007). These studies conclude that neuroinflammation accompanies early AD progression and blocking neuroinflammatory pathways might be beneficial in ameliorating tauopathies.

Hence, anti-inflammatory drugs have been used in clinical trials to prevent disease progression in AD. A study of nonsteroidal anti-inflammatory drugs (NSAIDS) in a Netherlands population (Rotterdam study) showed a significantly decreased risk of AD with increasing use of NSAIDS. In this study the short-term use of NSAID showed a relative risk of 0.95 while a long-term use of 24 months or more showed a relative risk of 0.2 with a confidence interval of 0.05–0.83 (In 't Veld et al., 1998). In a similar study reported in 2011 by Breitner et al. administration of NSAID Naproxen over a period of 2–3 years decreased the incidence of AD. These results were enhanced by measuring a marker of neurodegeneration, CSF ratios of tau to Aβ1-42 (Breitner et al., 2011). In contrast, a recent study by Marjerova et al. found that LPS treatment of immortalized microglial cells *in vitro* were capable of removing intracellular and extracellular tau oligomers by phagocytosis. These observations were validated *in vivo* in C57BL/6 mice. When injected with soluble and insoluble human tau aggregates, these mice displayed active microglial phagocytosis of both tau species (Barron et al., 2017). Thus, the suppression of immune system by anti-inflammatory drugs may not prove beneficial to AD treatment in the long run as these might enhance the spread of tau oligomers across healthy neurons.

### Inflammation in type 2 diabetes that influence neurodegeneration

Type 2 Diabetes has been associated with excess immune system activation, which increases the expression of proinflammatory cytokines especially microglia in the brain. It is noteworthy that Swaroop et al. observed elevated levels of TNFα, IL-1β, IL-2, and IL-6 in the hippocampus of diabetic animals (Swaroop et al., 2012). Previous studies have shown that incubation of cells with TNFα or high levels of FFA promotes inhibitory phosphorylation of the serine residues of IRS-1. This impairs the ability of IRS-1 to interact with the insulin receptor and generates an insulin-resistant condition capable of triggering Aβ and tau pathological cascades (Peraldi et al., 1996). It has also been demonstrated that obesity and hyperglycemia in T2DM contributes to ER and mitochondrial stress that generates reactive oxygen species (ROS). Elevated ROS then causes enhanced activation of inflammatory pathways (Kaneto et al., 2010; Back and Kaufman, 2012).

Along with evidences that relate oxidative stress and inflammation to the pathophysiology of diabetes, studies performed in various cellular and animal models suggest NFκβ activation is a key event early in the disease pathobiology and its complications. Several studies have shown that NF-κβ is induced by hyperglycemia and in conditions of neuronal damage (Romeo et al., 2002; Wellen and Hotamisligil, 2005). The activation of NF-κβ is followed by the expression of pro-inflammatory cytokines that jointly trigger brain inflammation and neuronal apoptosis eventually leading to cognitive decline. For instance, in the hippocampus of the STZ-treated rats there is a strong increase of ROS followed by NF-κβ activation (Locke and Anderson, 2011). Activated NF-κβ can induce cytotoxicity, trigger inflammation and promote apoptosis (Jaeschke et al., 2004). In STZ-induced retinopathy rat models, NF-κβ activation has been associated with enhanced expression of caspase 1 (Yin et al., 2017). Recent reports show that NF-κβ might be an important regulator of insulin sensitivity in T2DM by controlling the expression of GLUT2 receptor which is important for glucose secretion and transport in pancreatic beta cells (Patel and Santani, 2009).

Incidentally, the metabolic stresses that promote insulin resistance and T2DM also activate the inflammation and stress-induced kinases Ikβ kinase-β (IKKβ) and JUN N-terminal kinase (JNK) suggesting that these kinases play an important role in disease pathogenesis. Both JNK and IKκβ can phosphorylate the IRS-1 at the inhibitory Serine 307 thereby impairing insulin action (Jaeschke et al., 2004; Morel et al., 2010).

Oxidative stress in insulin resistance generates FFA and AGE products which result in glucotoxicity and impairment of insulin signaling. These ligands act through Toll-like receptors (TLRs) and receptors for advanced glycation end products (RAGE) that are also activated in neurodegenerative diseases (Ozcan et al., 2004). RAGE especially acts as a putative Aβ receptor and plays a significant role in AD pathogenesis. It is therefore predicted that the cumulative effect of these stress factors may lead to neuronal apoptosis and brain inflammation, both of which are prominent features of neurodegenerative diseases including AD.

To investigate whether inflammatory pathways act as a potential link between AD and T2DM, Takeda et al. generated a dual model of AD and T2DM by crossing an APP23 transgenic mice that overexpresses human APP to leptin-deficient ob/ob mice or polygenic NSY mice as a model for diabetes. The APP+/ob/ob dual transgenics showed increased levels of amyloid deposition around brain microvessels and enhanced cerebrovascular inflammation even before the manifestation of cerebral amyloid angiopathy. (Takeda et al., 2010). The authors also report an increased levels of RAGE in blood vessels as well as elevated levels of TNFα and IL-6 in the brain microvasculature of these animals. Progressive cognitive deficits and increased cerebrovascular inflammation were also noted in APP_+_-NSY dual transgenics raised on high-fat diet compared to NSY mice raised on the same diet. In a study conducted by Knight et al. a similar impact of high-fat diet was observed in 3xTg AD mice that overexpress triple mutations in human APP/MAPT-P301L/PSEN1 and in nontransgenic mice. When raised on a high-fat diet both the transgenics and the control animals displayed considerable weight gain and memory impairments. However, the memory impairments were more severe in the 3xTg mice compared to the controls. It is also interesting that although no significant differences were observed in the amyloid plaques and tau-tangle loads, the brains of 3xTg animals were accompanied by severe microgliosis that was not observed in age-matched controls. This strongly implies the role of neuroinflammation in the development of AD pathogenesis especially when subjected to abnormal dietary conditions (Knight et al., 2014).

Emerging studies have shown that loss of inflammatory mediators prevents insulin resistance, therefore pharmacological targeting of inflammatory pathways improve insulin action. For instance, salicylates activate insulin signaling by inhibiting inflammatory kinases within the cell (Kim M. S. et al., 2013). Similarly, targeting JNK using a synthetic inhibitor has been reported to enhance insulin signaling in obese mice and reduce atherosclerosis in ApoE mutant rodent model (Lee et al., 2003). There are yet other studies which show that thiazolidinediones (TZDs), high-affinity ligands of PPARγ act as insulin sensitizing agents and improve insulin action by activating lipid metabolism as well as by reducing the production of inflammatory molecules like TNFα (Peraldi et al., 1996).

These studies support a significant correlation between hyperglycemia, impaired insulin resistance, oxidative stress, and inflammation. All these factors are capable of directly impacting Aβ and tau pathologies as well as triggering a chain of inflammatory stress responses that might eventually lead to neurodegeneration as observed in AD.

## Autophagic impairments in AD and T2DM

Intracellular accumulation of misfolded protein aggregates is a salient feature of most neurodegenerative diseases (Frake et al., 2015). Autophagy is the process by which such protein aggregates are cleared from the neurons and is important for maintaining neuronal homeostasis. As neurons age they accumulate toxic intracellular protein aggregates and damaged organelles such as mitochondria that must be immediately cleared for the neuron to function at a physiological level (Lee, 2012; Son et al., 2012). Recent studies show that autophagic machinery is also involved in the pathophysiology of T2DM and it regulates the normal function of pancreatic beta cells. Insulin resistance generates oxidative stress on insulin-responsive tissues, enhanced autophagy in these cases acts as a protective factor (Masini et al., 2009). Other than indirect effects, there are studies that report direct impact of insulin resistance on autophagy by an inhibition of the downstream mTOR signaling pathway (Blagosklonny, 2013). Although, the connection between AD and T2DM pathogenesis in terms of autophagic dysfunction is not well documented, in this section we elaborate on the shared pathophysiologies of autophagy malfunctioning in these diseases and elaborate on the mechanisms by which insulin resistance might impact autophagy impairment and exacerbate AD pathogenesis.

### Autophagy malfunction in AD

**Figure 3:**
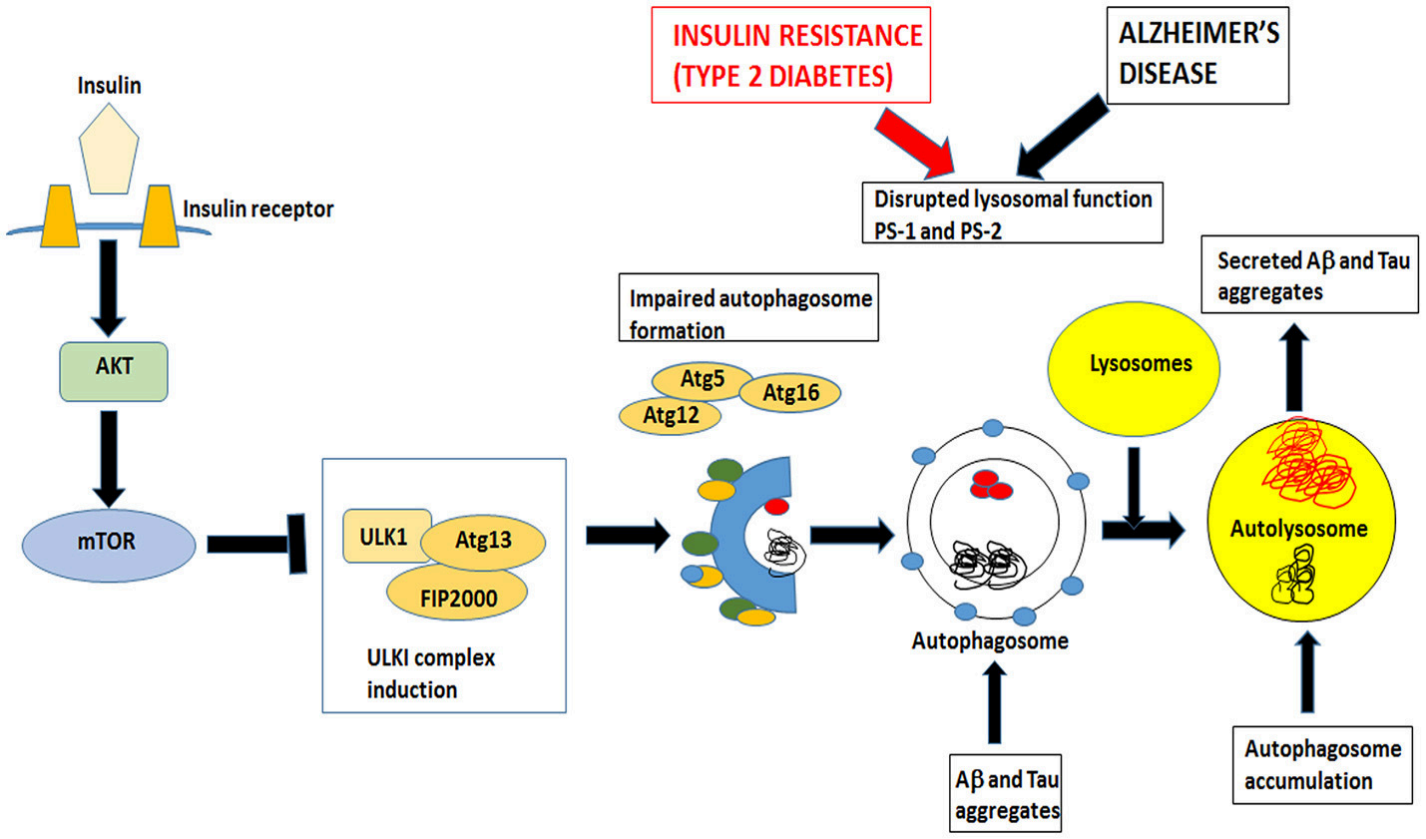
Insulin signaling also controls mTOR pathway that inhibits autophagy. Both insulin resistance in Type 2 Diabetes and Alzheimer's disease impairs the formation of autophagosomes and disrupts lysosomal function. Autophagosomes fuse with lysosomes to form autolysosomes. These autolysosomes have impaired lysosomal function in AD and T2DM and accumulate Aβ and tau aggregates. Undigested toxic aggregates are secreted out of the neurons and propagate toxic oligomers in adjacent neurons.

Macroautophagy is the most prevalent form of neuronal autophagy (Frake et al., 2015). In this process, cytoplasmic proteins and organelles are sequestered into double membrane bound structures called autophagosomes (Figure 3). In the next step, the autophagosomes fuse with the lysosomes to form autolysosomes or alternatively with endosomes to form amphisomes before fusing with lysosomes and finally the contents are degraded.

There is a substantial evidence that autophagy is dysregulated in the brains of AD patients. In 2005, Nixon and colleagues used immunogold labeling and electron microscopy techniques on AD brain biopsies of neocortical regions and detected diverse formations of immature autophagic vacuoles (AVs) in the dystrophic neurites (Nixon, 2007). The same phenomenon was also observed in transgenic animal models of AD. A study in PS-1/APP double transgenic mice showed that AVs were formed in dendrites and soma before the appearance of Aβ plaques compared to age-matched control animals (Nixon et al., 2005). Chen et al. using LC3-EGFP overexpressing 5X FAD mouse models and age-matched controls, observed increased accumulation of autophagosomes in the neurons of FAD-mouse models compared to the controls. This was more prominent under conditions of starvation. Interestingly, the macroautophagy induced by starvation in the transgenic animals was not sufficient to degrade the endogenous Aβ levels which resulted due to increased cellular uptake of extracellular Aβ (Chen et al., 2015). The importance of autophagy in the brain was highlighted in a study demonstrating that neuron-specific loss of autophagy proteins (ATG7 and ATG5) in mice results in neurodegeneration even when other pathological factors are absent (Frake et al., 2015). However, actual mechanisms underlying autophagic dysfunctions in AD has not been fully elucidated. Till date it is a matter of debate as to whether autophagy is the cause or a result of AD.

#### Presenilins as autophagy modulators

Neely et al. has shown that Presenilins play an important role in mediating autophagy as PS-1 has been shown to facilitate N-glycosylation of V0a1 subunit of the lysosomal vacuolar ATPase (v-ATPase) (Neely et al., 2011). FAD-associated mutations in PS-1 and PS-2 leads to an impairment of lysosomal function due to failed acidification of the internal lysosomal contents. This causes an increased accumulation of autophagosomes and a failure to fuse with dysfunctional lysosomes. In their study, Wilson et al. found that in PS-1–/– neurons both α and β synuclein are mislocalized to the lysosomes of the neuronal cell body and not in the presynaptic regions. The increased accumulation of synuclein suggests that PS-1 deficiencies play a crucial role in developing α-syn lesions in neurodegenerative diseases as observed in familial AD and PD (Wilson et al., 2004). Interestingly, Tung et al. found that non-neuronal and neuronal cells lacking PS-1 displayed reduced levels of p62 protein which serves as a “cargo receptor” for tau degradation. Their study suggests a novel mechanism by which the reduction PS-1 or its mutation in FAD impairs p62-dependent tau clearance (Tung et al., 2014).

#### Aβ as an autophagy modulator

There is a complex interplay between Aβ and autophagy. Several studies have shown that autophagy plays a crucial role in Aβ metabolism including Aβ production, secretion, and degradation (Nilsson et al., 2015). Autophagy facilitates the degradation and clearance of APP and all APP cleavage products comprising Aβ and APP-C-terminal-fragments. Deficiency of autophagy protein beclin 1 in cultured neurons and human APP transgenic mice resulted in elevated intraneuronal Aβ and formation of extracellular amyloid plaques. Overexpression of beclin 1 promoted neuronal autophagy, reduced Aβ levels, and ameliorated neurodegeneration (Pickford et al., 2008). Autophagy may also play a role in the secretion of Aβ into extracellular environment where it causes plaque formation. For instance, deletion of ATG7 in APP transgenic mice resulted in less amount of Aβ secretion and plaque formation (Xiong, 2015).

Interestingly, Aβ by itself could also be a regulator of autophagy as intracellular Aβ can activate autophagy by an AKT-dependent pathway or RAGE-CAMκβ-AMPK pathway by induction of mitochondrial ROS generation (Kim et al., 2017). Thus rapamycin, an mTOR inhibitor that upregulates autophagy, is able to reduce both Aβ pathology in AD mouse models and improve cognition (Spilman et al., 2010).

Several studies indicate that insulin resistance in T2DM inhibits the downstream mTOR pathway and activates autophagy. Insulin resistance also causes ER and oxidative stress that are capable of inducing autophagy (Quan et al., 2012). Ideally, enhanced autophagy should facilitate Aβ clearance; however, studies in ATG7 mouse models have shown that increased autophagy may also cause an increased secretion of Aβ in the extracellular matrix and enhance the deposition of Aβ plaques (Inoue et al., 2012). Thus, activation of autophagy under conditions of insulin resistance may worsen Aβ-mediated AD pathogenesis.

#### Tau and autophagic dysfunction

Recent studies suggest that autophagy plays a vital role in tau protein degradation and clearance (Inoue et al., 2012). A study in autophagy-impaired Nrf2 KO mice shows that the levels of phosphorylated and sarcosyl-insoluble tau increases (Jo et al., 2014). In ATG7 conditional KO mouse models the loss of ATG7 from the forebrains of transgenic mice leads to an accumulation of phospho-tau resembling pre-tangle formation within neurons (Inoue et al., 2012). On the restoration of autophagy, the levels of phospho-tau was found to be diminished. It has also been shown that the full-length tau (2N4R) and the caspase cleaved version (tauδC) are preferentially degraded by macroautophagy, while the truncated version of tau (taudelta280) is translocated to lysosomes by cell mediated autophagy (CMA) pathway (Dolan and Johnson, 2010).

Recent studies in primary neurons and transgenic P301S mouse models have demonstrated that treatment with autophagy-inducers trehalose and rapamycin reduced insoluble tau levels (Schaeffer et al., 2012; Ozcelik et al., 2013). Conversely, other studies have shown that mammalian target of rapamycin (mTOR) impairs tau clearance by inhibiting autophagy. The TSC1 and TSC2 are negative regulators of mTOR. Consequently, in TSC–/– transgenic mice the elevated levels of endogenous total and phosphorylated tau suggests an impairment of autophagy (Caccamo et al., 2013; Steele et al., 2013).

Under physiological conditions, tau promotes microtubule assembly and regulates microtubule dynamics (Janning et al., 2014). The microtubular arrays provide tracks for retrograde trafficking and maturation of autophagosomes before fusing with lysosomes in the soma (Kononenko, 2017). In the disease state, hyperphosphorylated tau is capable of disassembly and breakdown of microtubules that could subsequently inhibit retrograde trafficking causing the accumulation of immature autophagosomes within the axons (Rodríguez-Martín et al., 2013).

### Autophagic dysfunction in T2DM that can trigger AD pathogenesis

Insulin resistance in T2DM effectively results in increased oxidative stress that causes the production of ROS leading to damage of intracellular organelles such as ER and mitochondria (Jung et al., 2011). These ER and mitochondrial stress factors are the critical upstream events for the induction of downstream autophagic pathways for the removal and clearance of misfolded proteins in the ER lumen and the dysfunctional ER and mitochondria (Quan et al., 2012). However, during prolonged periods of intracellular stress autophagy pathway becomes inefficient leading to increased accumulation of autophagosomes and impaired clearance. *In vivo* studies in pancreatic beta-cell specific ATG7 KO mice have shown a decrease in the number of pancreatic beta-cells, impaired glucose tolerance, and reduction in insulin secretion (Chen et al., 2011). In addition, these cells accumulate large ubiquitinated proteinaceous materials and p62 implying an autophagic impairment. Supporting these observations, Fujitani et al. demonstrated an increased accumulation of autophagosomes in the pancreas of db/db mice (Fujitani et al., 2009). In addition, these ATG7-null beta-cells were found to be apoptotic leading to a decreased beta cell mass. When these ATG7 KO mice were crossed to ob/ob mice (a model for obesity with a mutation in the leptin gene) the progeny displayed severe diabetes suggesting that autophagic impairment in obese animals might make them more susceptible to diabetes (Quan et al., 2012). These studies suggest that autophagy acts as a neuroprotective factor in response to ER and mitochondrial stress generated in T2DM.

To elucidate the autophagic impairments underlying AD and T2DM, Jung et al. compared tau pathology and its associated signaling pathways in diabetic OLEF rats and age-matched non-diabetic controls (Jung et al., 2011). The scientists observed an increased accumulation of total and phospho-tau in the soluble fractions of brain extracts from OLEF rats. Interestingly, the increased accumulation of polyubiquitinated tau protein in the neurons of OLEF rats was accompanied by a decrease in p62 protein levels that is responsible for degradation of ubiquitinated tau by autophagy and proteasomal pathways In a similar study by Carvalho et al. using 3xTgAD and T2DM mouse models, a significant reduction was observed in the levels of autophagy markers ATG7 and LC3-II in the cerebral cortex and hippocampus of both these mice (Carvalho et al., 2015). Pronounced behavioral deficits were observed in these animals that correlated strongly with a reduction of the autophagy markers including the lysosomal marker LAMP1 suggesting an accumulation of autophagosomes and impaired protein clearance in both these models. It can be inferred that the impaired clearance of toxic, soluble aggregates of hyperphosphorylated tau protein is a critical mechanism underlying increased AD-like pathology in T2DM.

Taken together, these results show that firstly insulin resistance in T2DM is capable of inducing prolonged period of oxidative stress which leads to the failure of autophagic machinery and impaired autophagic clearance. This in turn may lead to the progressive build-up of toxic protein aggregates such as Aβ and tau oligomers and trigger AD pathogenesis. Secondly, under abnormal metabolic conditions or aging, autophagic impairment might be a crucial risk factor for T2DM. This could lead to a viscous cycle in which T2DM can promote tau hyperphosphorylation and induce the accumulation of autophagosomes within the neurons. Impaired autophagic clearance then triggers neurodegenerative events that lead to AD pathogenesis. The crucial role played by autophagy in AD and T2DM opens a new chapter in the development of pro-autophagy drugs that would be used as part of combinatorial therapy in targeting both diseases.

## Conclusion

In this review we have attempted to summarize the growing body of research that depicts the shared pathophysiology of AD and T2DM and elaborated on the underlying mechanistic pathways at the crossroads of these two diseases. However, it should be noted that almost all the animal models of Aβ and tau that have been used for preclinical studies are based on FTDP-17 cases and not on sporadic AD models. Although the mechanistic uderpinnings that link AD and T2DM could be similar, there is a possibility of considerable variation in the development and propagation of the disease pathology in the familial vs. sporadic cases. This is particularly relevant when designing combinatorial therapies.

A growing body of evidence suggests that the structural and functional integrity of the CNS is compromised in T2DM in the presence of excess insulin or under a condition of insulin resistance. In addition, T2DM impairs glucose metabolism and generates oxidative stress in vital cell organelles. Insulin resistance which is a prominent feature of T2DM is capable of increasing the production and secretion of Aβ by decreasing proteolysis by IDE. Also, insulin resistance dysregulates the PI3K/AKT/GSK-3β signaling cascade and generates hyperphosphorylated tau. Insulin resistance leads to loss of synapses, impaired autophagy and increased neuronal apoptosis. These alterations might trigger a cascade of events leading to abnormal Aβ and tau accumulation culminating in Alzheimer's disease pathology. Hence, targeting brain insulin signaling with pharmacological therapies used for treating T2DM is a novel and compelling approach to treat AD (Morris and Burns, 2012). This has given way to “drug-repositioning” strategies in which pre-existing anti-diabetic drugs are subjected to clinical trials to test their efficacy in AD therapeutics (Watson et al., 2005; Chen et al., 2009; Miller et al., 2011; Moore et al., 2013; Yarchoan and Arnold, 2014; Luchsinger et al., 2016; Femminella et al., 2017).

## Author contributions

SC researched articles and prepared the manuscript for the review. AM critically revised the draft before submission.

### Conflict of interest statement

The authors declare that the research was conducted in the absence of any commercial or financial relationships that could be construed as a potential conflict of interest.

## Footnotes

^1^Available online at: https://www.alz.co.uk/research/WorldAlzheimerReport2015.pdf.
